# The Dark Side of the Self-Determination Theory and Its Influence on the Emotional and Cognitive Processes of Students in Physical Education

**DOI:** 10.3390/ijerph16224444

**Published:** 2019-11-12

**Authors:** Rubén Trigueros, José M. Aguilar-Parra, Remedios López-Liria, Patricia Rocamora

**Affiliations:** 1 Department of Psychology, Hum-878 Research Team, Health Research Centre, University of Almería, 04120 Almería, Spain; rtr088@ual.es; 2 Department of Nursing, Physiotherapy and Medicine, Health Research Centre, University of Almería, 04120 Almería, Spain; rll040@ual.es (R.L.-L.); rocamora@ual.es (P.R.)

**Keywords:** teacher psychological control, psychological need frustration, emotional intelligence, meta-cognitive strategies, physical education

## Abstract

Amongst the main objectives of physical education (PE) classes is the consolidation of healthy lifestyle habits in young people and adolescents. Nonetheless, these classes can also provide the basis from which adverse experiences are generated which affect students’ perceptions of these classes. Previously conducted studies have focused on motivational processes and not on emotional processes, nor on the way in which students learn. The objective of the present study was to explore the dark side of the self-determination theory, its influence on emotional intelligence and the meta-cognitive strategies of students. Methodology: A total of 1602 young people undertaking secondary education participated, with self-reported ages between 13 and 19 years. The following questionnaires were utilized: *Controlling Coach Behaviors Scale, Frustration of Psychological Needs in PE classes Scale, Emotional Intelligence in PE Scale and Motivated Strategies for Learning Questionnaire.* A structural equation model was developed which explained causal associations between the study variables. Results: Psychological control positively predicted each one of the sub-factors of frustration of psychological needs. Frustration of psychological needs negatively predicted emotional intelligence. Finally, emotional intelligence positively predicted meta-cognitive thinking. Conclusions: The influence and importance of the teaching style adopted by teachers is indicated, in addition to the effect of students’ psychological experiences on emotions and learning strategies.

## 1. Introduction

Despite the physical, psychological, and emotional benefits provided by regular engagement with physical activity (PA), only 20% of adolescents worldwide participate in a habitual way [1]. For this reason, Physical Education (PE) classes can contribute to resolving this problem, given that one of its fundamental objectives is the adoption of healthy lifestyle habits, such as engagement with regular extra-curricular PA [2]. However, for PE students to internalize these habits and the benefits they produce, it is necessary that pupils commit information to memory in an ordered way. This favors information recovery and means it can be applied within other similar contexts. This aspect is known as learning transfer [3]. Current studies principally focus on the motivational processes of students and their transfer towards PA [4]. Specifically, the self-determination theory (SDT) has been applied to the positive aspects of psychological processes, neglecting the negative aspects. In this sense, it is indicated that PE classes may also generate the adoption of maladaptive behaviors by students, above all when faced with unpleasant experiences [5]. Further, few studies exist that have considered the influence of emotions and the learning strategies employed by students during PE classes. For this reason, the present study seeks to explore the influence of the SDT on emotional intelligence and meta-cognitive strategies, taking a more critical perspective.

The SDT [6] suggests that the social context can influence individuals through the presence of two very different interpersonal styles within it. One of these is autonomy supportive, whilst the other is highly controlling. The first of these marks the beginning of the light perspective. It refers to teacher promotion of personal initiative, self-regulation and self-concept in students, with all of these making minimum use of contingencies [7]. On the other hand, the controlling style marks the beginning of the dark side. It refers to the application of external pressures by teachers and the use of coercive means, impositions, etc. These are perceived by students and form the origin of their behaviors, undermining their personal initiative, effort, and personal self-knowledge [8]. In this way, teachers will influence the psychological need development of their students, with the type of influence depending on the role they choose to adopt [9].

According to the SDT, psychological needs are basic and essential nutrients for development and personal wellbeing [10]. In this sense, there are three psychological needs: Autonomy (the extent to which an action carried out by an individual comes from their own interests), competence (the feeling of ability and of executing a task to a certain level), and relatedness with others (the feeling of ability and of executing a task to a certain level) [11]. Further, a study conducted by González-Cutre, Sicilia, Sierra, Ferriz, and Hagger [12] proposed the incorporation of novelty as a psychological need. This is defined as the innate tendency of an individual to seek new activities and experiences in order to achieve complete development and wellbeing. In this way, students who feel more autonomous when they participate in decision making feel supported and integrated within their reference social group, and will also be more competent when they carry out relevant actions. Those who consider the activities they engage in to be different and engaging will experience psychological need satisfaction. This represents the normal path when considering SDT from the light perspective. It is related with the learning of new abilities, commitment to learning, improved interpersonal relationships and engagement in adaptive behaviors [6]. In exchange, outcomes will be negative if, during PE classes, students experience feelings of abandonment, limited success from their actions, lack participation in decision making and judge actions to be excessively monotone or repetitive. These students will instead experience frustration of their psychological needs, this being the route understood by the dark side. This is related with abandonment of the activity, lack of commitment, a deficit in interpersonal relationships and, ultimately, manifestation of maladaptive behaviors [13].

Multiple studies have analyzed the influence of teachers and of the psychological needs of students from the tenants of the SDT, in the educational context of PE [4,14,15]. However, the majority of these studies have taken their focus from the light side of the SDT, with few studies available that have conducted their analysis taking the dark side. This discrepancy is even more evident if only studies conducted in the Spanish context are considered. In this sense, one study carried out by Baena-Extremera, Gómez-López, Granero-Gallegos, and Martínez-Molina [16] analyzed the extent to which autonomy acted as a predictor of enjoyment, learning climate, and the psychological need of autonomy in secondary school students. In the same way, Taylor and Lonsdale [17] showed that autonomy supportive teachers positively influenced the satisfaction of psychological needs, effort, and vitality in students during PE classes. Further, Zhang, Solmon, Kosma, Carson, and Gu [18] concluded that autonomy support was positively associated with satisfaction of the psychological needs (autonomy, competence, and relatedness with others), intrinsic motivation and intention to be physically active. Taking the dark side, a study conducted by Haerens, Aelterman, Vansteenkiste, Soenens, and Van Petegem, [19] demonstrated that teacher control negatively impacted upon satisfaction of the psychological needs (autonomy, competence, and relatedness with others) and autonomous motivation. In contrast, it positively influenced frustration of psychological needs and controlling forms of motivation. On the other hand, Trigueros et al. [15] have shown that when teachers exert psychological control in PE classes, enjoyment, trust, and motivation of students are negatively impacted. Further, De Meyer [20] confirmed that the controlling role of teachers positively predicted controlled forms of motivation and demotivation, and negatively impacted autonomous motivation.

Given the interest shown in these issues, the present study seeks to analyze the influence of the controlling role of teachers on frustration of the four psychological needs (autonomy, competence, relatedness with others, and novelty), taking the dark side of the SDT. This is crucial given that there is a lack of studies that have analyzed this issue. The line of work opened by González-Cutre et al. [12] and Trigueros et al. [15] will set the basis of the study. In addition, the study wishes to deepen knowledge about the influence of frustration of the four psychological needs on emotional intelligence, as previous research has barely considered the emotions experienced by students during PE classes, and much less recognition of these emotions. It has been concluded that the area of PE specifically contributes to the development of social and emotional intelligence due to the fact that it provides a conducive environment to the transmission of feelings and emotions that humanize personal contact through motor activities [21].

Emotional intelligence (IE) is understood as a skill that facilitates the recognition and regulation of emotions and supports the generation of adaptive behaviors [22]. However, multiple theories have been developed to help better understand the concept of IE, with two in particular standing out: The trait model [23] and the ability model [24]. Both theories have various elements in common, such as the fact that emotions are considered as predictors of positive adaptive behaviors [25]. Nevertheless, differences lie in the fact that the trait theory considers IE as a construct that is linked to the set of stable personality traits, socio-emotional competencies, motivational aspects, and diverse cognitive skills that are essential for overcoming demands and pressures [23]. In exchange, the ability model considers IE as another form of intelligence that is based on the adaptive use of emotions and their application to thinking, and facilitates adaptation of the individual to the environment and problem solving.

According to various studies in the educational context, IE has been positively associated with better psychological wellbeing in students [26], emotional wellbeing [25], academic performance, social relationships [27], self-efficacy and empathy [28]. On the other hand, emotional intelligence has been negatively associated with stress [26], depression [29], and negative emotions [30]. All of these are elements that can lead to the generation of maladaptive behaviors.

Following this thread, the present study seeks to analyze within the context of PE, the extent to which emotional intelligence is related with meta-cognitive strategies. In this sense, meta-cognitive strategies could provide a useful tool to help students assimilate information from the exterior via their own knowledge and skills, or those that they are now able to acquire [31]. To this end, emotional intelligence can exert a significant influence upon meta-cognitive strategies given that recognition and control of one’s own emotions largely determines decision making [32].

**Figure 1:**
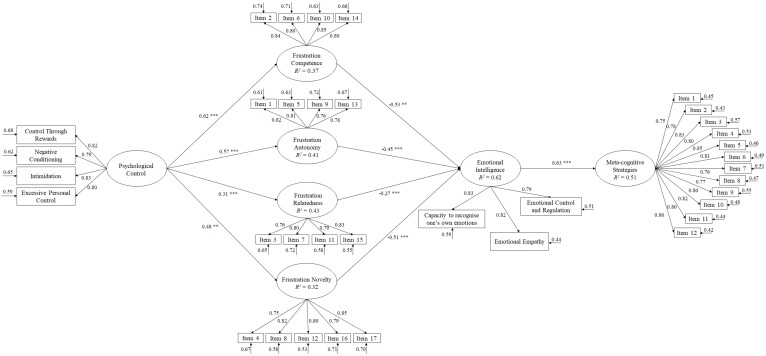
Of structural equations showing the relationships between the different variables. All parameters are standardized and statistically significant. The variances explained are shown above the small arrows. Note: ** *p* < 0.001; ** *p* < 0.01.

Bearing these considerations in mind, the present study was designed in which the following hypotheses were proposed (see Figure 1): (1) Teacher control will positively predict the frustration of psychological needs and the frustration of novelty; (2) frustration of the four psychological needs (autonomy, competence, novelty, and relatedness with others) will positively predict emotional intelligence; and (3) emotional intelligence will positively predict meta-cognitive strategies.

## 2. Method

### 2.1. Participants

The sample was formed by 1602 secondary school students (820 males and 882 females), with ages reported between 13 and 19 years (M = 15.73; SD = 1.30). All participants came from the province of Almeria. Non probabilistic convenience sampling was employed as a function of the educational centers and students that could be accessed.

### 2.2. Instruments

Controlling style. The Spanish version developed by Trigueros et al. [33] and adapted from the Controlling Coach Behaviors Scale (CCBS) of Bartholomew, Ntoumanis, and Thogersen-Ntoumani [34] was used. This questionnaire comprised 15 items divided between four factors, which measured control through rewards, negative conditioning, intimidation, and excessive personal control. The questionnaire was responded to via a Likert scale that runs from 1 (totally disagree) to 7 (totally agree).

Frustration of psychological needs: The Frustration of Psychological Needs in PE classes Scale of Trigueros, et al. [13] was used. The scale comprises 17 items divided between autonomy, competence, relatedness with others, and novelty. The questionnaire is headed by the phrase “During PE classes…” and is responded to via a Likert scale that runs from 1 (totally disagree) to 7 (totally agree).

Emotional Intelligence. The Spanish version of the Emotional Intelligence in PE Scale of Cecchini et al. [25] was used. This scale was developed from the version of Arruza et al. [35]. The questionnaire comprises 22 items divided between three factors, which measure the capacity to recognize one’s own emotions, emotional control and regulation, and emotional empathy. The questionnaire is responded to via a Likert scale that runs from 1 (totally disagree) to 7 (totally agree).

Learning focus. The Motivated Strategies for Learning Questionnaire (MSLQ; [36]) which was validated and adapted into Spanish by Roces, Tourón and González [37] was used with the purpose of measuring strategies of meta-cognition. Only the 12 items that refer to metacognition strategies were used. Students responded using a Likert scale that ranged from 1 (not at all true) to 5 (totally true).

### 2.3. Procedure

Firstly, permission was requested from the Bioethical Committee for Human Research of the University of Almeria (Ref. UALBIO 2019/014) with the aim of contacting various educational centers in the province of Almeria. After informing the educational centers about the objectives of the study, the parents or legal guardians signed informed consent as the pupils were underage. Scales were administered to participants. This was done under the supervision of the study’s principal researcher, who provided instructions and resolved any doubts that arose during questionnaire completion. The time taken to complete the questionnaire was estimated at around 25 min.

### 2.4. Data Analysis

Initially, descriptive statistical analysis (mean and standard deviation) was conducted through *Pearson* correlation, carrying out correlational analysis between all study variables. Next, internal consistency was examined (Cronbach alpha) with the purpose of testing reliability of the factors integrated within the study. Following this, the hypothesized predictive model was tested via a structural equation model (MEE).

For the MEE, the maximum likelihood estimation model with bootstrapping was conducted using the statistical package AMOS 20. In order to judge the model tested, various fit indices were considered: χ^2^ /gl, CFI (Comparative Fit Index), IFI (Incremental Fit Index), RMSEA (Root Mean Square Error of Approximation) alongside its associated confidence interval (IC) al 90%, and SRMR (Standardized Root Mean Square Residual). Values of *χ^2^/gl* lower than 3, values for the incremental indices (CFI, IFI) equal to or greater than 0.95, and RMSEA and SRMR values lower than or very close to 0.06 and 0.08, respectively, were considered to indicate adequate fit of the model to the study data [38]. However, Marsh, Hau, and Wen [39] state that these cut-points must be interpreted with caution as they have been shown to be excessively restrictive and difficult to achieve when complex models are tested.

## 3. Results

### 3.1. Descriptive Statistics, Reliability Analysis, and Bivariate Correlations

As shown in Table 1, participating students obtained a mean score for emotional intelligence and meta-cognitive strategies of 4.95 and 3.22, respectively. Analysis of internal consistency revealed Cronbach alpha values that were greater than 0.80 for each of the variables. Correlation analysis showed that those factors that are linked to the dark side of the SDT were positively correlated amongst themselves, whilst being negatively related to emotional intelligence and meta-cognitive strategies. In contrast, the relationship between emotional intelligence and meta-cognitive strategies produced a negative correlation.

### 3.2. Structural Equation Model Analysis

For the hypothesized model of predictive relationships (Figure 1), fit indices were shown to be adequate: *χ^2^* (551, N = 1602) = 1774.90, *χ^2^/gl* = 3.22, *p* < 0.001, IFI= 0.96, CFI = 0.96, RMSEA = 0.052. (IC 90% = 0.047 − 0.061), SRMR = 0.038. These results were adjusted to established parameters, thus, the proposed model can be accepted as demonstrating adequate fit [39].

Following this, the relationships obtained between the different factors integrated in the model were described (Figure 1):

(1) Teachers’ psychological control positively predicted frustration of each one of the psychological needs: Autonomy (β = 0.54, *p* < 0.001), competence (β = 0.63, *p* < 0.001), relatedness with others (β = 0.52, *p* < 0.01) and novelty (β = 0.33, *p* < 0.001).
(2) Frustration of autonomy negatively predicted emotional intelligence (β = −0.41, *p* < 0.001); frustration of competence negatively predicted emotional intelligence (β = −0.52, *p* < 0.01); frustration of novelty negatively predicted emotional intelligence (β = −0.29, *p* < 0.001); and finally, frustration of relatedness with others negatively predicted emotional intelligence (β = −0.43, *p* < 0.001).
(3) Emotional intelligence predicted meta-cognitive strategies (β = 0.60, *p* < 0.001) in a positive way.

## 4. Discussion

The dark side of the SDT was analyzed (this is to say, psychological controlling and frustration of psychological needs) with regards to its influence on emotional intelligence and meta-cognitive strategy use in secondary school students in the area of PE. Studies up to this moment with PE students have focused only on the light side of the SDT, analyzing the effect of autonomy support, psychological need satisfaction and motivation at a cognitive, physical and social level [4]. In addition, the present study contemplates for the first-time various forms of intelligence in students. This is seen through the fact that being capable of recognizing emotions and knowing how to control them can lead to a series of adaptive behaviors. This includes paying attention to information is one’s surroundings [40]. On the other hand, as a novel aspect, the present study centered on an analysis of meta-cognitive strategies. In this sense, the majority of previously conducted studies had observed the motivational processes of students [11] but had failed to indicate how they process information, or their capacity to code and retain it.

Diverse studies in the PE setting, have discussed the positive effect of autonomy support in relation to psychological need satisfaction in PE students and the effect of this, in turn, on motivation towards PE classes [6,41]. However, there are barely any research studies available that have considered the negative aspects also present during PE classes. Such aspects can include the controlling style of the teacher and frustration of psychological needs. Depending on the role of teachers and the perception of students regarding the PE classes in which they participate, such aspects can exert a negative influence over the adoption of adaptive behaviors, both in the present and in the future. In this sense, previous research studies have indicated, on occasion, that low scores for autonomy support and for psychological need satisfaction, are predictors of controlling behavior and frustration, respectively [34]. However, this statement is not totally adequate given that the items of the scale, these being both autonomy support and psychological need satisfaction, only collect information regarding positive experiences. In this way, it is unlikely that negative aspects of these experiences would emerge [42].

Results of the present study showed that perceived control positively predicts frustration of each one of the psychological needs (e.g., autonomy, competence, and relatedness with others), in addition to novelty frustration. These results are comparable to those reported by the few other conducted studies, at both a national and international level, where it has been demonstrated that psychological controlling could be positively related with frustration of psychological needs [43,44]. Thus, these results are along the same lines as those presented by previous studies and fall within the tenants of the SDT. The results highlighted that the relationships between psychological controlling, psychological need frustration and novelty, can explain to a certain extent the likely outcomes for students who perceive a lack of control over their own decisions and teachers who behavior in an autocratic, restrictive, or pressurizing way towards students. It is explained that they will feel overwhelmed, incapable and rejected, due to them feeling that their psychological needs are being frustrated. Further, when teachers work within their comfort zone, attempt to innovate little and offer the same experiences during their classes, students will experience novelty frustration.

It has been shown that each one of the factors relating to psychological need frustration and novelty frustration, negatively predict emotional intelligence. This data can be compared to that of other studies conducted in other fields. In this sense, a study carried out from the light perspective of the SDT showed that psychological need satisfaction acted as a predictor of the emotional wellbeing of students [45]. In addition, Cordeiro, Paixão, Lens, Lacante, and Sheldon [46] indicated that psychological need satisfaction acted as a positive predictor of psychological and emotional wellbeing. In contrast, frustration of psychological needs acted as a negative predictor of these aspects. Further, results of the present study show agreement with the tenants of the SDT [6], given that psychological need frustration can trigger a series of maladaptive consequences such as disinhibition and/or engagement in behaviors that go against personal wellbeing. This is supported by outcomes of a study carried out by Balluerka, Gorostiaga, Alonso-Arbiol, and Aritzeta [47], which showed high levels of emotional intelligence in students to act as predictors of psychological and emotional wellbeing.

As a consequence, it is necessary that PE teachers work with students throughout the full duration of the course to develop their emotions and control, with the aim of becoming conscious of these emotions and thus, being able to work towards a greater level of wellbeing [19]. Further, it is also fundamental that PE classes are motivational and interesting so that students can take away positive lived experiences that will favor their learning.

Finally, the present results showed that emotional intelligence acts as a positive predictor of meta-cognitive strategies. The conclusions are similar to those drawn by Hasanzadeh and Shahmohamadi [48] with university students enrolled on various degree programs. These authors also showed a positive relationship between emotional intelligence and the use of meta-cognitive strategies. In this sense, Villavicencio and Bernardo [49] state that emotions act as a link between sensory information and thought. In this way, when experiences are interpreted in a positive way we feel motivated to act and to achieve objectives. In contrast, when experiences are interpreted in a negative way we do not act. This creates a maladaptive and counter-productive behavioral pattern, which impedes learning and stunts emotional/mental growth.

Finally, it is highlighted that the present research shows support for the tenants of the SDT, taking the dark side, introducing new variables and demonstrating their applicability in Spanish culture. The model seems to show good robustness and capacity for generalization towards different cultures or ages. It helps us to better understand the role played by the teacher in the cognitive and emotional processes of students, in addition to lending a deeper understanding of learning strategies. However, as one of the limitations of the present study, it must be indicated that it deals with a correlational study design which does not permit causal relationships to be identified. Further, the results obtained can be interpreted in a number of ways depending on the understanding of a given individual. To this end, the model sought to present different probabilities with the purpose of explaining existing relationships between study variables. In this way, future research studies should conduct a deeper analysis of the results achieved through intervention studies in order to better clarify the relationship between the different variables. In addition, future studies should analyze the differences in relation to the emotional state and motivation towards the Physical Education classes of the adolescents according to their country of residence. Finally, it would be interesting to understand the influence of motivation and the concrete emotional state of each individual according to age, given the variability young people suffer as they grow and start to become more autonomous in their decision making, in addition, the possibility of analyzing the pressure exerted by the social context on young people’s decision-making as they grow should not be overlooked.

## Figures and Tables

**Table 1:** Descriptive statistics, internal consistency analysis and bivariate correlations.

Factors	*M*	*DT*	Range	α	1	2	3	4	5	6	7
1. Psychological Control	1.82	1.06	1–7	0.82		0.47 ***	0.48 **	0.50 ***	0.49 ***	−0.52 ***	−0.45 ***
2. Frust. Autonomy	2.12	1.47	1–7	0.83			0.68 ***	0.57 ***	0.65 ***	−0.42 **	−0.36 ***
3. Frust. Competence	2.14	1.32	1–7	0.88				0.70 ***	0.71 **	−0.40 ***	−0.33 ***
4. Frust. Relatedness with others	1.96	1.33	1–7	0.81					0.70 ***	−0.46 **	−0.38 **
5. Frust. Novelty	1.88	1.34	1–7	0.87						−0.47 ***	−0.41 ***
6. Emotional Intelligence	4.95	1.51	1–7	0.92							0.76 ***
7. Meta-Cognitive Strategies	3.22	1.12	1–5	0.83							

*** *p* < 0.001; ** *p* < 0.01. Note: M = Mean; SD = Standard deviation; α = Cronbach alpha; Frust = Frustration.
